# High-Energy and High-Power Pseudocapacitor–Battery Hybrid Sodium-Ion Capacitor with Na^+^ Intercalation Pseudocapacitance Anode

**DOI:** 10.1007/s40820-020-00567-2

**Published:** 2021-01-08

**Authors:** Qiulong Wei, Qidong Li, Yalong Jiang, Yunlong Zhao, Shuangshuang Tan, Jun Dong, Liqiang Mai, Dong-Liang Peng

**Affiliations:** 1 Department of Materials Science and Engineering, Fujian Key Laboratory of Materials Genome, College of Materials, Xiamen University, Xiamen, 361005 People’s Republic of China; 2 State Key Laboratory of Advanced Technology for Materials Synthesis and Processing, Wuhan University of Technology, Wuhan, 430070 People’s Republic of China; 3 Shenzhen Geim Graphene Center, Tsinghua Shenzhen International Graduate School, Tsinghua University, Shenzhen, 518055 People’s Republic of China; 4 Advanced Technology Institute, University of Surrey, Guildford, Surrey GU2 7XH UK

**Keywords:** Sodium-ion capacitors, Pseudocapacitance, Hybrid capacitors, Two-dimensional materials, Iron vanadate

## Abstract

High-performance and low-cost sodium-ion capacitors (SICs) show tremendous potential applications in public transport and grid energy storage. However, conventional SICs are limited by the low specific capacity, poor rate capability, and low initial coulombic efficiency (ICE) of anode materials. Herein, we report layered iron vanadate (Fe_5_V_15_O_39_ (OH)_9_·9H_2_O) ultrathin nanosheets with a thickness of ~ 2.2 nm (FeVO UNSs) as a novel anode for rapid and reversible sodium-ion storage. According to in situ synchrotron X-ray diffractions and electrochemical analysis, the storage mechanism of FeVO UNSs anode is Na^+^ intercalation pseudocapacitance under a safe potential window. The FeVO UNSs anode delivers high ICE (93.86%), high reversible capacity (292 mAh g^−1^), excellent cycling stability, and remarkable rate capability. Furthermore, a pseudocapacitor–battery hybrid SIC (PBH-SIC) consisting of pseudocapacitor-type FeVO UNSs anode and battery-type Na_3_(VO)_2_(PO_4_)_2_F cathode is assembled with the elimination of presodiation treatments. The PBH-SIC involves faradaic reaction on both cathode and anode materials, delivering a high energy density of 126 Wh kg^−1^ at 91 W kg^−1^, a high power density of 7.6 kW kg^−1^ with an energy density of 43 Wh kg^−1^, and 9000 stable cycles. The tunable vanadate materials with high-performance Na^+^ intercalation pseudocapacitance provide a direction for developing next-generation high-energy capacitors.

**Electronic supplementary material:**

The online version of this article (10.1007/s40820-020-00567-2) contains supplementary material, which is available to authorized users.

## Highlights

- Layered iron vanadate ultrathin nanosheets (FeVO UNSs) with a thickness of ~ 2.2 nm were synthesized by a sonicate-assisted method.
- Pseudocapacitive Na^+^ intercalation of FeVO UNSs anode delivers high initial coulombic efficiency (93.86%), high reversible capacity (292 mAh g^−1^), excellent rate capability, and remarkable cycling stability.
- A pseudocapacitor–battery hybrid SIC is assembled with the elimination of presodiation and delivers high energy and power densities.

**Electronic supplementary material:**

The online version of this article (10.1007/s40820-020-00567-2) contains supplementary material, which is available to authorized users.

## Graphical abstract

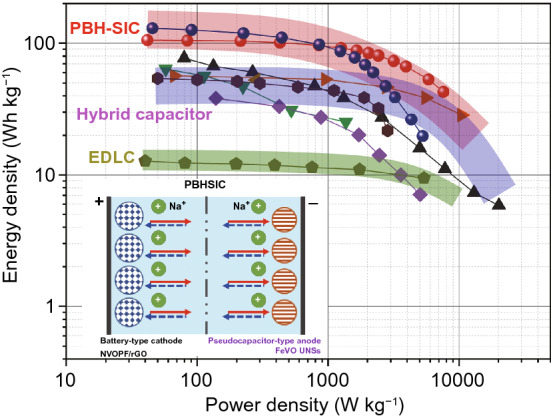

## Introduction

Electrochemical energy storage (EES) devices, such as batteries and supercapacitors, power the portable electronics and electric vehicles that are indispensable parts of our daily lives [1, 2]. The high-energy batteries and high-power supercapacitors derive from different mechanisms, leading to the different charge-storage properties and application fields [2–5]. Importantly, there are imperious requirements of new EES devices combining both high energy and high power densities, as well as at low cost [6]. Lithium-ion capacitors (LICs), consisting of an electronic double-layer capacitor (EDLC)-type cathode (e.g.*,* activated carbon or graphene) and a faradaic-type anode (*e.g.,* Li_4_Ti_5_O_12_, Nb_2_O_5_, or prelithiated graphite), are successful examples that deliver both high power and energy densities [2, 7–9]. However, they face the increase in cost owing to limited lithium resource. Alternatively, sodium resource is very abundant, making the sodium-ion capacitors (SICs) are very promising candidates for large-scale applications [2–4, 10–15]. Unfortunately, Na^+^ has larger radius (1.02 Å) than Li^+^ (0.76 Å), leading to the non-intercalated reactions, or sluggish intercalation/extraction kinetics and accompanied large volume variations [10, 16, 17]. As a result, most of the sodium-ion storage anode materials exhibited low reversible capacity, poor rate capability, and limited cycling stability, which were inferior to those of lithium-ion ones [2, 10]. Therefore, there is a clear need for novel sodium-ion storage anode materials, delivering high-rate capability (up to 100 C) in appropriate potential range (before the electrolyte decompose), to enable fast charging and long-period high-power delivering for high-performance SICs.

Pseudocapacitance, including surface redox reaction and bulk fast ion intercalation, shows faradaic charge storage process without diffusion limitation, enabling the delivery of high capacity at high rate [1, 4]. The pseudocapacitive Li^+^ storage materials have been proved and delivered ultrahigh rate performance [1, 6, 18]. The pseudocapacitive Na^+^ storage was lack of well study. Decreasing the size of the active material into nanoscales is one of the most investigated routes to boost pseudocapacitive reaction [1, 6]. Especially, with the high surface area and aspect ratios, the two-dimensional (2D) nanosheets (e.g., MXene) provide largely exposed redox-active sites and overcome the diffusion-controlled kinetic limitations [19–22]. Recently, sodium-ion storage anode nanomaterials and nanocomposites, such as Ti_2_CT_*x*_-MXene nanosheets [19–21], mesoporous TiO_2_/reduced graphene oxide (rGO) [23], nano-Nb_2_O_5_@C/rGO [24], and MoS_2_ nanosheets [25, 26], showed high-rate capability owing to the contribution of pseudocapacitance. However, their initial coulombic efficiency (ICE), specific capacity, high-rate capability, cycling stability, and safety needed to be further optimized [8, 19–28]. Most of the anode materials operated at low potential (0.01–1 V vs. Na^+^/Na) usually accompanied with electrolyte decomposition, thick solid electrolyte interphase (SEI) layers formation [16], and irreversible phase transformations [19, 25], resulting in a low ICE (the charge–discharge curves of some anode materials are shown in Fig. S1) and the risk of metal dendrite growth at high rate. Besides, the ion diffusion kinetics was extremely restricted by passing through thick SEI layers [16], blocking the high-rate delivery. Additionally, the low ICE anode materials needed to be presodiated before assembling into SICs, which is complex, ineffective, and costly [23, 24, 26]. Furthermore, the traditional hybrid capacitors were hardly to reach the further goal of an energy density over 100 Wh kg^−1^, owing to the use of non-Faradaic and low-capacity EDLC-type electrodes (see the detailed discussion in Fig. S2) [1]. Therefore, the next-generational SICs need to be high energy and power densities that utilize faradaic reaction on both cathode and anode sides, thousands of cycles, and easy-assembled at low cost. But it remains largely unexplored.

Vanadates include various types of tunnel and layered materials for sodium-ion storage, such as the Zn_0.25_V_2_O_5_·*n*H_2_O nanobelts [29] and MnV_2_O_6_ nanosheet [30]. However, these materials still operated at low potentials, which displayed low ICE and unsatisfactory rate capability [31]. Iron vanadates are nature existence, abundant, and low cost [32]. The iron and vanadium are both redox reactive, offering multi-electron reactions for delivering very high capacity [33–35]. For example, porous Fe_2_VO_4_ microparticles anode delivered a sodiation capacity of 601 mAh g^−1^ in a large potential range of 0.01–3 V vs. Na^+^/Na, which followed the conversion reaction mechanism [35]. Recently, it was found that the layered iron vanadate with a large spacing displayed high reversibility for sodium-ion intercalation at high potential [29, 34]. According to the electrochemical property of iron vanadates [32–35], their redox reaction potential could be tuned in an appropriate range through optimizing the nanosized morphology, stoichiometric ratios, crystal structure, and element valances. It is hypothesized that the optimization of vanadate as anode material is able to be as an appropriate example with high ICE, high specific capacity, and high-rate capability for easy-assembled high-energy SIC. But the electrochemical performance and detailed charge storage mechanism have not been reported yet.

Herein, we develop a facile and high-yield ultrasonic treatment method to synthesize the Fe_5_V_15_O_39_(OH)_9_·9H_2_O ultrathin nanosheets (named as FeVO UNSs). The high surface area of ultrathin FeVO UNSs provides a large number of exposed redox-active sites to the electrolyte, promoting the diffusion kinetics. Based on various in situ electrochemical measurements including in situ and ex situ synchrotron high-energy X-ray diffraction (XRD), ex situ scanning electron microscope (SEM), and electrochemical kinetics analysis, we demonstrate that the FeVO UNSs anode shows a single-phase reaction during Na^+^ intercalation/extraction through tuning the reaction potential window. The Na^+^ intercalation/extraction is the pseudocapacitive-dominated process, displaying excellent rate capability and cycling performance. Furthermore, benefiting from the pseudocapacitive FeVO UNSs anode, a novel pseudocapacitor–battery hybrid SIC (PBH-SIC) is assembled that free from any additional presodiation process on the anode side and delivers an excellently high energy density, high power density, and thousands of stable cycles.

## Experimental Section

### Synthesis of Fe_5_V_15_O_39_(OH)_9_·9H_2_O UNSs

The FeVO UNSs were prepared by a facile water bath method [33] combined with an additional sonicate treatment. Briefly, NH_4_VO_3_ (12 mmol) was dissolved in 200 mL deionized water at 70 °C. The FeNO_3_·9H_2_O (4 mmol) was dissolved in 40 mL deionized water. Then, the FeNO_3_ solution was dropwise added into NH_4_VO_3_ solution with stirring. The mixed solution was hold at 100 °C for 6 h. The final brown color precipitates were washed by centrifugation with deionized water and ethanol for each two times. Then, the precipitates were dispersed in deionized water and strongly sonicated for thirty minutes. The sonicated suspension was centrifugated at 3000 rpm for 5 min for removing the precipitations. Then, the supernatant liquid was centrifugated again at 10,000 rpm for ten minutes for obtaining the precipitations of FeVO UNSs. Finally, the FeVO UNSs were obtained after drying in vacuum oven at 100 °C for 12 h.

### Synthesis of Na_3_(VO)_2_(PO_4_)_2_F/rGO Composites

Graphene oxide (GO) aqueous dispersion was prepared using the well-known Hummers method. The Na_3_(VO)_2_(PO_4_)_2_F/reduced GO (NVOPF@rGO) was prepared by the one-pot solvothermal method [36]. First, NH_4_VO_3_, NaF, and NH_4_H_2_PO_4_ in a molar ratio of 2:3:2 were dissolved in 20 mL GO dispersion (2 g L^−1^) under stirring. Then, 20 mL *N*,*N*-dimethylform amide was slowly added to the above mixture with stirring for 1 h. The resulting solution was transferred into a 50-mL Teflon-lined autoclave and heated at 180 °C for 24 h. Afterward, NVOPF/rGO was washed three times with deionized water and ethanol and then dried in a vacuum oven at 100 °C for 12 h.

### Material Characterization

The morphologies were characterized by scanning electron microscope (SEM, JOEL 7100F) and transmission electron microscope (TEM, Titan G2 60-300). The synchrotron-based X-ray diffraction was carried out at Beamline 13-BM-C of the Advanced Photon Source (APS), Argonne National Laboratory, and the wavelength was 0.434 Å. In situ synchrotron high-energy XRD during the first sodiation process was carried out at 11-ID-C beamline of the APS, while the wavelength was 0.11725 Å. A custom-designed coin cell with a MACCOR cycler was discharged from OCV to 0.8 V at a specific current of 0.1 A g^−1^. The XRD patterns were collected every 10 min using a PerkinElmer 2D X-ray detector, during cell operating. The nitrogen absorption/desorption measurements were taken on a Tristar-II 3020 instrument at 77 K. The atomic force microscopic (AFM) measurement was characterized by using Bruker MultiMode 8 Atomic Force Microscope. Inductively coupled plasma (ICP) test was performed on the PerkinElmer Optima 4300DV spectrometer.

### Electrochemical Measurements

Coin cells (CR2032) were assembled in an Ar-filled glove box (O_2_ and H_2_O levels less than 1 ppm). The composite weight ratio of FeVO UNSs: conductive carbon: binder (mixed carboxyl methyl cellulose (CMC) and styrene butadiene rubber (SBR) in a ratio of 1:1 by weight) is 75:15:10. The NVOPF/rGO electrode was made by mixing with conductive carbon, polyvinylidene fluoride (PVDF) in 1-methyl-2-pyrrolidinone (NMP) in the weight ratio of 82:10:8. Then, the homogeneous slurries were coated onto the carbon-coated Al foil and dried in vacuum oven at 120 °C for 12 h. Then, the film was cut into disks (a diameter of 10 mm). The mass loading of the FeVO UNSs and NVOPF/rGO is controlled at 1.5–2.0 and 4.0–5.0 mg cm^−2^, respectively. For half-cell testing, sodium metal (a diameter of 14 mm and thickness of ~ 1 mm) was used as the count and reference electrode, Celgard-2350 as the separator, 1 M NaPF_6_ (30 μL) in diethylene glycol dimethyl ether as the electrolyte. For assembling the FeVO UNSs//NVOPF/rGO sodium-ion capacitor, the mass ratio was calculated by *m*_+_*C*_+_ = *m*_−_*C*_−_. Usually, ~ 5% excessive capacity on anode side was considered; therefore, the calculated ratio of m_+_: m_−_ ≈ 2.50. Galvanostatic charge/discharge rate performance and cyclic voltammetry were performed by using Bio-Logic VMP3 potentiostat at room temperature. The long-term cycling performance was performed by using NEWARE battery testing system (CT-4008). The specific energy density and average power density are calculated according to Eqs. (1) and (2):

1 $$E= {\int }_{0}^{t}IV \mathrm{d}t$$

2 $${P}_{\mathrm{ave}}= \frac{E}{t}$$

where *I* (A g^−1^) is the constant current density, *V* (V) is working voltage, and *t* (h) is the time of a discharge process.

## Results and Discussion

### Synthesis and Characterizations of the FeVO UNSs

**Fig. 1:**
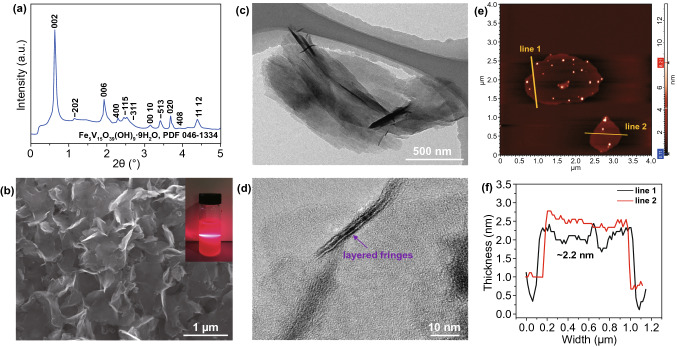
Characterizations of the FeVO UNSs. **a** Synchrotron XRD pattern of FeVO UNSs. **b** SEM, **c** TEM, **d** HRTEM images of FeVO UNSs, **e** AFM image and **f** associated height profiles of FeVO UNSs

The FeVO UNSs were synthesized by a facile water bath method [33] combined with an additional sonicate treatment to exfoliate the nanoflowers (Fig. S3) into separated UNSs (Fig. 1b). The synthesis could be easily scale up in a high yield. The synchrotron high-energy XRD pattern shows that the FeVO UNSs exhibit the well crystalline diffraction peaks (Fig. 1a). All the peaks can be indexed to a pure phase of Fe_5_V_15_O_39_(OH)_9_·9H_2_O (JCPDS No. 46–1334, monoclinic, *a* = 11.84 Å, *b* = 3.65 Å, *c* = 21.27 Å, *β* = 100°) [34]. The ICP results show that the atomic ratio of Fe and V is 1.00:3.04, which is very close to the stoichiometric ratio. The photograph of well-dispersed FeVO UNSs in water (inset of Fig. 1b) shows the obvious Tyndall effect. SEM (Fig. 1b) and TEM (Fig. 1c) images show the uniformity of FeVO UNSs with a width of ~ 1 μm. High-resolution TEM (HRTEM) image (Fig. 1d) displays an edge of curved nanosheet with large layered fringes. The average thickness of ~ 2.2 nm for a single nanosheet was measured by AFM (Fig. 1e, f). The Brunauer–Emmett–Teller (BET)-specific surface area (SSA) of FeVO UNSs is 62.6 m^2^ g^−1^ (Fig. S4), which is higher than that of FeVO flowers (34.3 m^2^ g^−1^).

### Sodium-ion Storage Mechanism of FeVO UNSs Anode

For a layered anode material, the sodiation reaction usually divides into two steps: intercalation and followed conversion reaction. Their boundary is mainly dependent on the cutoff reaction potential [25]. Half cell (2032-type coin cell) was assembled to measure the electrochemical performance. It is the first time that the FeVO is regarded as anode material for sodium-ion storage. At the beginning, cyclic voltammetry (CV) scanned at different cutoff potentials was measured to determine the boundary before the occurrence of irreversible reaction. The upper limited potential was set at 3.4 V, according to the open-circuit voltage (OCV) of 3.38 ± 0.03 V (vs. Na^+^/Na). The CV curves in different potential windows are shown in Fig. S5. In the potential range of 0.8–3.4 V, the CV curves show box shape with broad cathodic peaks at 2.16 and 1.40 V and anodic peak at 1.93 V, which are typical pseudocapacitive behaviors [18]. However, lowering the cutoff potential below 0.8 V, the redox peaks are gradually disappearing (Fig. S5), and this is an irreversible process (Fig. S6).

**Fig. 2:**
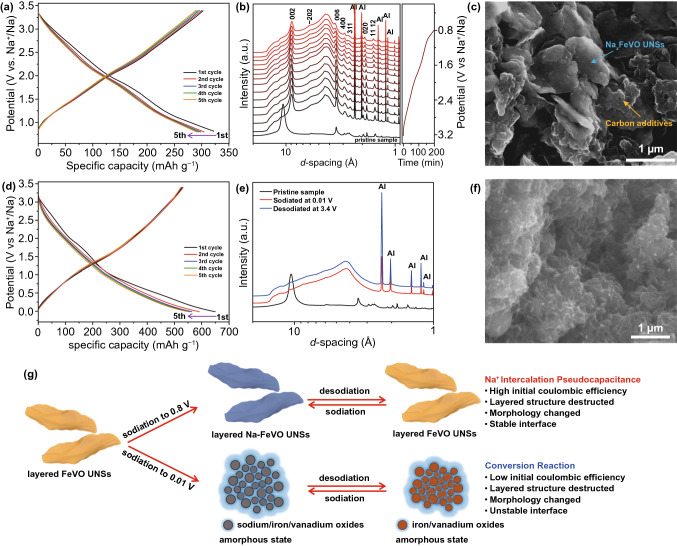
Sodium storage mechanism of FeVO UNSs anode. **a** Charge–discharge curves cycled at 0.1 A g^−1^ in 0.8–3.4 V. **b** In situ synchrotron XRD patterns during sodiation to 0.8 V. **c** Ex situ SEM image after cycled in 0.8–3.4 V. **d** Charge–discharge curves cycled at 0.1 A g^−1^ in 0.01–3.4 V. **e** Ex situ synchrotron XRD patterns after sodiation at 0.01 V and de-sodiation at 3.4 V. **f** Ex situ SEM image after cycled in 0.01–3.4 V. **g** Schematic of sodium-ion storage mechanism for FeVO UNSs in different potential windows

The galvanostatic (GV) charge and discharge of hall cells were measured at 0.1 A g^−1^ in the potential windows of 0.8–3.4 and 0.01–3.4 V, respectively. Cycled in 0.8–3.4 V, the FeVO UNSs anode shows quasi-linear-like galvanostatic curves (Fig. 2a), consistent with the CV results. It delivers a high initial sodiation and desodiation capacity of 322 and 303 mAh g^−1^, respectively. The corresponding ICE is 93.86%, and the coulombic efficiency quickly climbs to 99.30% in 5 cycles (Fig. S7a). The FeVO UNSs anode delivers a reversible sodiation capacity of 292 mAh g^−1^ (corresponding to a capacitance of 404 F g^−1^) at the 5^th^ cycle. The discharge and charge curves between the first and the following cycles are well overlapped, indicating highly reversible reaction process at the first sodiation step.

To figure out the sodium-ion storage mechanism and the interface reaction chemistry, the in situ and ex situ synchrotron XRD patterns and ex situ SEM images were collected. Figure 2b shows the in situ synchrotron high-energy XRD patterns during discharging to 0.8 V. The *x*-axis of XRD patterns is conversed into *d*-spacing, which clearly shows the layer spacing changes. The *d*_002_ decreases from 10.63 to 8.81 Å at the beginning, owing to the enhanced layered interaction by the intercalated sodium ions [37]. During the sodiation, the diffraction peaks of FeVO well-maintained without the generation of new peaks indicate a solid solution reaction process. The *d*_002_ is slightly increased to 8.99 Å and then dropped back to 8.80 Å. When the FeVO UNSs anode was de-sodiated at 3.4 V (ex situ XRD results in Fig. S8), the *d*_002_ remains at 8.81 Å. Owing to the remain of Na^+^ ions in between the layers after desodiation, the *d*_002_ does not shift back to the pristine state. The small interlayer spacing shifts during Na^+^ intercalation and extraction mean slight volume changes, which is beneficial for long-term reversible cycles. Comparing to the morphology of pristine FeVO UNSs anode (Fig. S9), the ex situ SEM image (Fig. 2c) confirms the retention of nanosheet morphology after cycled in 0.8–3.4 V.

When cycled in the large potential range of 0.01–3.4 V, the FeVO UNSs anode shows a low ICE of 81.41% and unstable CE from ~ 90 to 95% in the following 4 cycles (Figs. 2d and S7b). The corresponding XRD patterns (Fig. 2e) show that the crystal phase turns into amorphous state after totally sodiated at 0.01 V, and it does not recover when desodiated back to 3.4 V. Meanwhile, ex situ SEM image (Fig. 2f) shows the disappearance of nanosheet morphology and the aggregation of thin nanoparticles. The loss of crystalline and change of morphology are the typical behaviors of conversion reaction with rapid fading of redox reversibility [38].

**Fig. 3:**
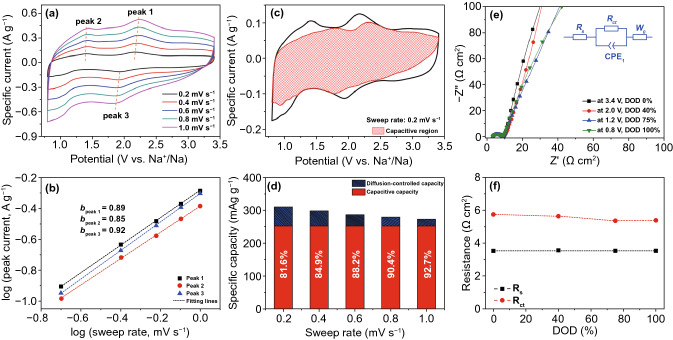
Kinetics analysis of pseudocapacitive FeVO UNSs anode. **a** CV curves of FeVO UNSs anode in 0.8–3.4 V at different sweep rates. **b** Logarithm peak current *vs.* logarithm sweep rate plots to determine the *b*-value of redox peaks. **c** Separation of capacitive contribution (shaded area) at a sweep rate of 0.2 mV s^−1^. **d** Related capacitive and diffusion-controlled capacity at different sweep rates, indicating a pseudocapacitive-dominated charge storage process. **e** Operando time-lapse Nyquist plots of FeVO NSs cycled in 0.8–3.4 V at different DOD states. **f** Plots of *R*_s_ and *R*_ct_ at different DOD states

Based on the results above, a detailed sodium-ion storage mechanism of FeVO is schematically displayed in Fig. 2g. By limiting the cutoff potential at 0.8 V, the FeVO UNSs anode undergoes reversible sodium ion intercalation with single-phase changes. This phenomenon is different from those of MoS_2_ with 2H-to-1 T irreversible phase transformation [25] or Ti_2_CT_*x*_-MXene with an initial irreversible activation process [19]. This sodium-ion intercalation process is pseudocapacitive-dominated, as discussed at kinetics analysis section (Fig. 3) [39]. The crystallographic layer structure of FeVO UNSs is very stable during the sodiation processes with slightly “lattice-breathing effect” (Fig. 2b). The operation potential above 0.8 V vs. Na^+^/Na is safe, which inhibits the electrolyte decomposition and thick SEI formation, leading to the high ICE and safety [1, 7, 40]. The FeVO anode experiences a conversion reaction in 0.01–3.4 V, exhibiting low ICE and poor cycling stability (Fig. S10).

### Intercalation Pseudocapacitance-Dominated Kinetics Analysis

The kinetics analysis was based on a series of CV measurements at various sweep rates [41]. Figures 3a and S11 show the stepwise CV curves of FeVO UNSs electrode under the sweep rates of 0.2–1 and 2–10 mV s^−1^, respectively. The redox peaks shift slightly with the increased sweep rates, indicating rapid reaction rates. The relation of the measured current (*i*) and scan rate (*v*) can be inferred from Eq. (3) [41]:

3 $$i = av^{b}$$

where *b* is determined by the slope of log (*i*) vs. log (*v*) plots for redox peaks. The *b* = 0.5 indicates diffusion-controlled process, while *b* = 1 means capacitive charge storage process. The *b* values of three redox peaks were calculated as 0.89, 0.85, and 0.92, respectively (Fig. 3b), suggesting that this particular redox process follows a mixture of diffusion-controlled and capacitor-like responses upon reduction, while the pseudocapacitive reaction is dominated.

The total charge storage can be divided into capacitive- and diffusion-controlled contribution parts, which can be quantitatively analyzed by Eq. (4) suggested by Dunn and co-workers [42]:

4 $$i\left( V \right) = k_{1} v + k_{2} v^{1/2}$$

where *k*_1_*v* represents capacitive responds and *k*_2_*v*^1/2^ is the diffusion-controlled process. In this case, the quantitative analysis was calculated at the slow rate of 0.2 mV s^−1^, when the diffusion-controlled process was maximized [1]. Figure 3c shows a CV curve for the capacitive content, delivering a dominated capacitive contribution of 81.6%. As sweep rate increased, the value of capacitive contribution is enlarged to 92.7% at 1 mV s^−1^ (Fig. 3d). These results indicate the pseudocapacitive-dominated sodium-ion storage behavior, which is beneficial for delivering fast charge and discharge rates [40].

Operando electrochemical impedance spectra (EIS) at different depth of discharge (DOD) states were collected to analyze the electrochemical behavior of FeVO UNSs anode [43]. The Nyquist plots (Fig. 3e) show no noticeable changes during sodiation from 3.4 to 0.8 V. The low-frequency straight lines show an extremely high phase angle from the states of DOD 0–100%, which is a general electrochemical feature for pseudocapacitive materials [4]. Figure 3f shows the stable quantitative resistances of electrolyte solution (*R*_s_) and *R*_ct_ at ~ 3.5 and ~ 5.5 Ω cm^2^, respectively, indicating the highly stable electrolyte–electrode interface [44].

### Electrochemical Performance of FeVO UNs Anode

**Fig. 4:**
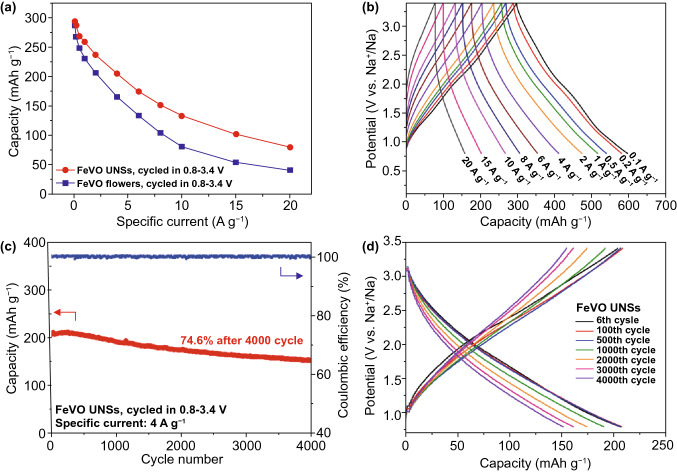
Electrochemical performance. Rate capability of FeVO UNSs and FeVO flowers, cycled in 0.8–3.4 V. **b** Charge–discharge profiles of FeVO UNSs at different specific currents. **c** Cycling performance of FeVO UNSs at 4 A g^−1^ and **d** related charge–discharge profiles at different cycles

The rate performance was measured, as shown in Fig. S12a–c. At first, the rate capability of the FeVO UNSs and FeVO flowers (Fig. S2, the SSA is 34.3 m^2^ g^−1^) is compared in Fig. 4a. At the slow specific current, both the two samples deliver closed capacity of ~ 290 mAh g^−1^, indicating that all the active sites are available for faradaic sodium storage. However, at high currents, the capacity of FeVO UNSs exhibits much higher than that of FeVO flowers (80 *vs.* 36 mAh g^−1^ at 20 A g^−1^). The enhancement of high rate capability is owing to the ultrathin feature of FeVO UNSs that effectively provide more exposed surface-active sites for faradaic adsorption of Na^+^ ions at fast rates. Figure 4b shows the charge–discharge curves of FeVO UNSs exhibiting linear slope at each high specific current. The ultrafast and stable of pseudocapacitive reaction is rather better than that of conversion reaction at high rates (Fig. S12d), even the latter is operated in a wider potential window.

Long-term cycles at a high specific current of 4 A g^−1^ were measured, while they were preactivated at 0.1 A g^−1^ for five cycles (Fig. 4c). The sodiation capacity of FeVO anode cycled in 0.8–3.4 V is 205 mAh g^−1^, and it maintains at 153 mAh g^−1^ after 4000 cycles, indicating a capacity retention of 74.6%. The related charge–discharge curves (Fig. 4d) exhibit the linear slops within all the cycles. The excellent electrochemical performance of the FeVO UNSs with high ICE, ultrahigh rate capability, superior reversibility, and safe operation potential are outstanding in comparison with other state-of-the-art anodes (Table S1) [19, 20, 23, 24, 39, 45–51].

### Pseudocapacitor–battery Hybrid Sodium-ion Capacitor

**Fig. 5:**
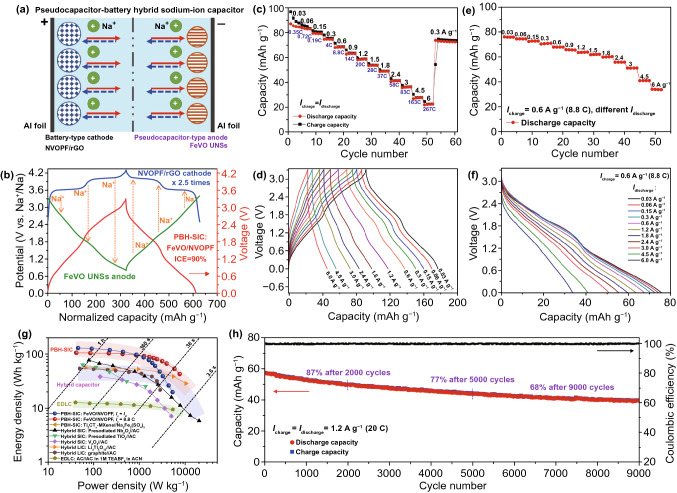
Electrochemical performance of PBH-SIC. **a** Schematic of PBH-SIC consisting of pseudocapacitive FeVO UNSs anode and battery-type NVOPF/rGO cathode. **b** Charge–discharge profiles of NVOPF/rGO cathode, FeVO UNSs, and the assembled SIC. **c** Rate capability and **d** related charge–discharge curves of PBH-SIC, when the charge and discharge currents are equal. Holding the charging rate at 0.6 A g^−1^ (8.8 C), the rate capacity (**e)** and related discharge voltage profiles (**f**) at various discharging currents. **g** Ragone plots of FeVO//NVOPF PBH-SIC and the reported state-of-the-art hybrid SICs and LICs. **h** Ultralong-term cycling performance the PBH-SIC at 1.2 A g^−1^ (20 C)

The pseudocapacitive FeVO UNSs is a promising anode material for SICs. To further enlarge the energy density of SICs, we propose a novel pseudocapacitor–battery hybrid SIC (PBH-SIC), as schematically displayed in Fig. 5a. The Na-rich battery-type cathode provides a large amount of de-intercalated Na^+^ ions to be stored at anode side, similar to “rocking-chair” batteries. The battery-type Na_3_(VO)_2_(PO_4_)_2_F (NVOPF) cathode [36, 52, 53] has been proved to deliver high-rate capability; thus, it was selected to assemble the PBH-SIC. The NVOPF/rGO composites were synthesized by a modified solvothermal method (Fig. S13) [36]. The NVOPF/rGO cathode (Fig. S14) delivered a high reversible capacity of 126 mAh g^−1^, two high operation plateaus at ~ 3.6 and 4.1 V, fast rate capability up to 20 A g^−1^ (with a capacity of 22 mAh g^−1^), and long-term cycling stability (a capacity retention of 99.6% after 800 cycles at 1 A g^−1^).

Figure 5b shows the related charge–discharge curves. The NVOPF cathode provides extraction of Na^+^ into FeVO UNS anode at the charge step. Then, the Na^+^ moves contrary at discharge step. The galvanostatic charge and discharge curves of the NVOPF//FeVO PBH-SIC show capacitive quasi-linear slopes (red line in Fig. 5b) in a voltage range of 0 to 3.3 V. Benefiting from the high ICE of the FeVO anode, the assembled PBH-SIC displays a high ICE of ~ 90% at the matched mass ratio of cathode to anode ≈ 2.5:1. Here, the specific current, capacity, energy, and power were calculated based on the mass of total cathode and anode materials. The PBH-SIC delivers a high discharge capacity of 87 mAh g^−1^ at 0.03 A g^−1^ (equal to 0.35 C, a rate of 1C corresponds to complete charge or discharge in one hour) [44] and excellent high-rate performance (Fig. 5c). The reversible capacity of ~ 58, 41, and 23 mAh g^−1^ is obtained at 1.2 (20 C), 2.4 (58 C), and 6 (267 C) A g^−1^, respectively, when the charge and discharge currents are equal. The related charge–discharge curves (Fig. 5d) show the typical capacitor-like triangular shapes at each specific current. Additionally, PBH-SIC exhibits excellent fast-charging and high-power delivering performance (Fig. 5e, f). When maintained at the fast charge rate of 8.8 C, the PBH-SIC could functionally deliver an enhanced capacity of 34 mAh g^−1^ at 6 A g^−1^.

According to the integration of galvanostatic curves, the discharge energy density and power density were calculated, as shown in the Ragone plots (Fig. 5g). The PBH-SIC exhibits a maximum energy density of 126 Wh kg^−1^ at a power density of 91 W kg^−1^. At the high power density of 1.3, 2.2, and 5.2 kW kg^−1^, the delivered energy density is 87, 60, and 20 Wh kg^−1^, respectively. When the charge rate is hold at 8.8 C (0.6 A g^−1^), the output power ability is much enlarged, displaying a high power delivering up to 7.6 kW kg^−1^ with 43 Wh kg^−1^.

It is worth noting that the long-term stability of this PBH-SIC (Figs. 5h and S15) was cycled at the high rate of 1.2 A g^−1^ (20 C), while the related DOD is 67%. The capacity retention is 68% after 9000 cycles. The superior cycling stability is benefited from highly stable pseudocapacitive FeVO UNSs anode with robust lattice and morphology (Fig. 2b, c), the stable electrode–electrolyte interface (Fig. 3e), and the stable NVOPF/rGO cathode (Fig. S14c). The ex situ SEM image of FeVO anode in cycled PBH-SIC (Fig. S16) shows the maintaining of UNSs morphology without thick SEI formations, indicating high safety and high-rate long-term usage. Based on the same calculation method by accounting the total weight of cathode and anode materials, our presodiation-free PBH-SIC delivers much higher energy and power densities than those of previous reported state-of-the-art hybrid capacitors, even those including an additional presodiation process on anode side (Fig. 5g and Table S2) [19, 23, 27, 54–56].

## Conclusions

Two-dimensional FeVO UNSs are synthesized by a high-yield ultrasonic treatment method. The FeVO UNSs anode delivers highly reversible pseudocapacitive Na^+^ intercalation in optimized reaction potential range. Confirmed by in situ synchrotron XRD and electrochemical analysis, the sodium-ion intercalation/extraction of FeVO UNSs anode undergoes no phase change process with robust layered structure and morphology. As a result, the FeVO UNSs anode delivers high ICE of 93.86%, a reversible specific capacity of 292 mAh g^−1^, superior rate capability (80 mAh g^−1^ at 20 A g^−1^), and excellent cycling stability (4000 cycles). In addition, the FeVO UNSs anode is used for developing a novel PBH-SIC, in which complex and unsafe presodiation processes are waived. Remarkably, the assembled FeVO//NVOPF PBH-SIC delivers a high specific energy of 126 Wh kg^−1^ (at 91 W kg^−1^) and power density of 7.6 kW kg^−1^ (with an energy density of 43 Wh kg^−1^), as well as 9000 stable cycles. This work provides a fundamental insight into the design of tunable vanadate material with Na^+^ intercalation pseudocapacitance that delivers high-rate capability. The successfully presodiated-free assembled PBH-SIC resolves the major scientific and engineering bottleneck of SICs, which is very promising for developing next-generation low-cost EES devices with both high energy and power densities.

## Electronic supplementary material

Below is the link to the electronic supplementary material.

Supplementary material 1 (PDF 1872 kb)
