# Supplementary material for: High-Energy and High-Power Pseudocapacitor–Battery Hybrid Sodium-Ion Capacitor with Na+ Intercalation Pseudocapacitance Anode
Source: Nanomicro Lett. 2021 Jan 8;13:55. doi: 10.1007/s40820-020-00567-2 (PMC8187546; doi:10.1007/s40820-020-00567-2)
Supplement: Supplementary file 1 — Supplementary material 1 (PDF 1872 kb) [file 40820_2020_567_MOESM1_ESM.pdf]

Supporting Information for

High-Energy and High-Power Pseudocapacitor-Battery Hybrid Sodium-Ion Capacitor with Na^+ Intercalation Pseudocapacitance Anode

Qiulong Wei[1,] *, Qidong Li[3], Yalong Jiang[2], Yunlong Zhao[4], Shuangshuang Tan[2], Jun Dong[2], Liqiang Mai[2,] *, Dong-Liang Peng[1,] *

[1]Department of Materials Science and Engineering, Fujian Key Laboratory of Materials Genome, College of Materials, Xiamen University, Xiamen 361005, P. R. China

[2]State Key Laboratory of Advanced Technology for Materials Synthesis and Processing, Wuhan University of Technology, Wuhan 430070, P. R. China

[3]Shenzhen Geim Graphene Center, Tsinghua Shenzhen International Graduate School, Tsinghua University, Shenzhen 518055, P. R. China

[4]Advanced Technology Institute, University of Surrey, Guildford, Surrey GU2 7XH, United Kingdom

*Corresponding authors. E-mail: qlwei@xmu.edu.cn (Qiulong Wei); mlq518@whut.edu.cn (Liqiang Mai); dlpeng@xmu.edu.cn (Dong-Liang Peng)

Supplementary Figures and Tables

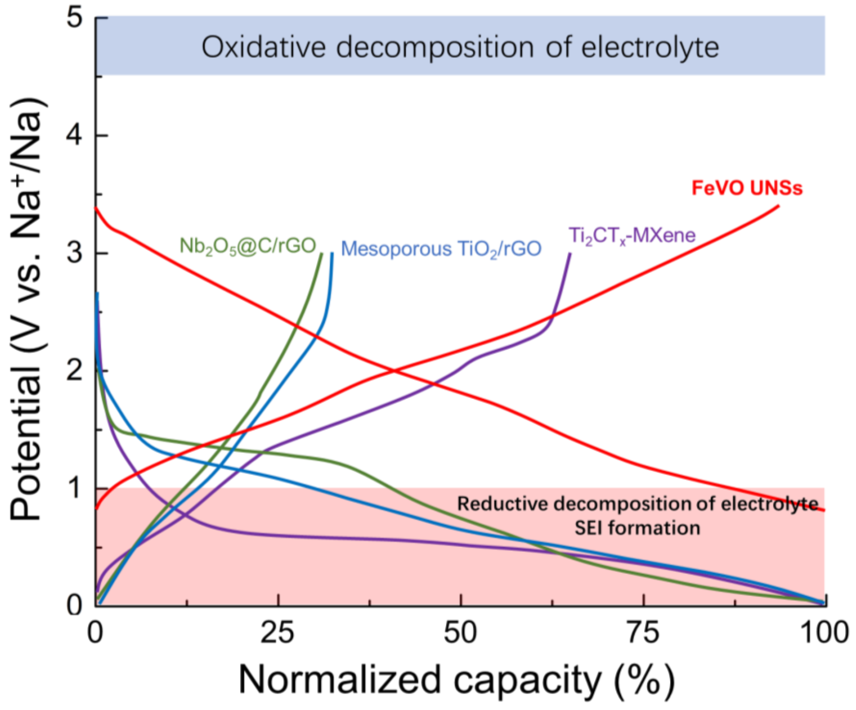

Fig. S1 Initial charge-discharge profiles of mesoporous TiO_2/rGO (Ref. [S1]), nano-Nb_2O_5@C/rGO (Ref. [S2]), Ti_2CTx-MXene nanosheet (Ref. [S3]) and the as-synthesized FeVO UNSs anode

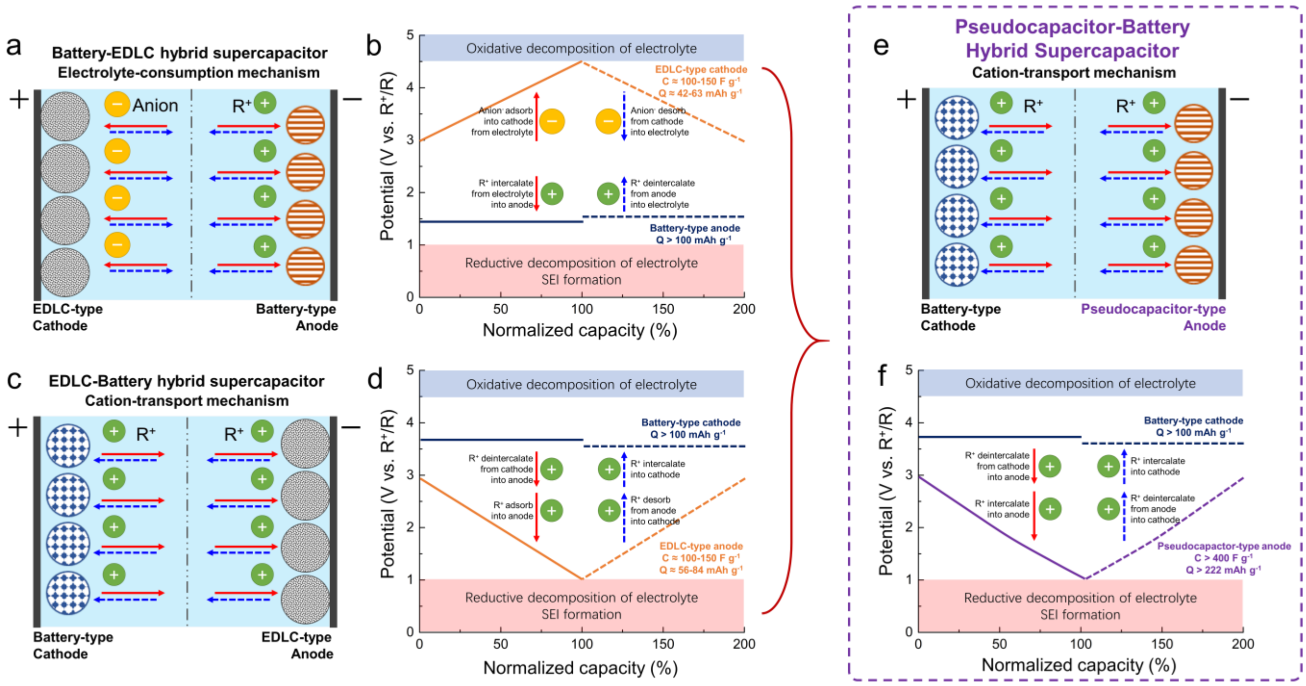

Fig. S2 Schematic of hybrid supercapacitors. (**a, b**) Battery-EDLC hybrid supercapacitor, consisting of battery-type anode and EDLC-type cathode. The charge carriers (both cations and anions) come from the electrolyte, identified as electrolyte-consumption mechanism. (**c, d**) EDLC-Battery hybrid supercapacitor, consisting of EDLC-type anode and battery-type cathode. The charge carriers are based on cations from cathode then adsorbed by anode, identified as cation-transport mechanism. (**e, f**) Proposed of Pseudocapacitor-Battery hybrid (PBH) supercapacitor, consisting of pseudocapacitor-type anode and battery-type cathode. PBH supercapacitor follows cation-transport mechanism: the charge carriers are based on cations extraction from cathode then into pseudocapacitive anode. The both electrodes of PBH supercapacitor are with faradaic reaction can largely enhanced the energy density. Note: R^+ represents Li^+ or Na^+, solid line represents a charge process, and dashed line represents a discharge process.

Figure S2a, b shows the well-known model of hybrid supercapacitor consisting of battery-type anode and EDLC-type cathode (*e.g.*, activated carbon (AC)), known as Battery-EDLC hybrid supercapacitor. All the charge carriers (both cations and anions) come from the electrolyte, which is identified as electrolyte-consumption mechanism [S4]. For example, the hybrid lithium-ion capacitor (LIC), utilizing $Li_4Ti_5O_{12}$ anode (170 mAh g^{-1} with a plateau at 1.55 V *vs.* Li^+/Li) coupled with AC cathode (the potential of zero charge for AC cathode is 3.08 V *vs.* Li/Li^+), delivered an energy density of ~55 Wh kg^{-1} [S5]. Figure S2c, d display another type of hybrid supercapacitor which consists of EDLC-type anode and battery-type cathode, named as EDLC-Battery hybrid supercapacitor. All charge carriers are based on cations from cathode moving into anode, identified as cation-transport mechanism [S4]. For example, the hybrid LIC consisting of high-rate $LiFePO_4$ cathode (170 mAh g^{-1} with a plateau at 3.4 V *vs.* Li^+/Li) matched with AC anode exhibited an energy density of 43.3 Wh kg^{-1} [S6]. However, the above reported EDLC-battery or battery-EDLC hybrid models were hardly to reach the further goal of developing next-generational hybrid supercapacitor with an energy density over 100 Wh kg^{-1}, owing to the use of EDLC-type electrodes with very low capacity. For example, the commercialized AC (*e.g.*, YP50F) only delivered a specific capacitance of ~100-150 F g^{-1} in non-aqueous electrolyte, corresponding to a specific capacity of ~42-63 mAh g^{-1} in 3-4.5 V or ~56-84 mAh g^{-1} in 3-1 V (calculated based on the equation of $Q = C \times \Delta V$) [S7].

Remarkably, pseudocapacitive anode could deliver a specific capacitance of >400 F g^{-1} (corresponding to the specific capacity >222 mAh g^{-1}), which are much higher than that of EDLC-type electrode. Therefore, the pseudocapacitor-battery hybrid (PBH) supercapacitor (Fig. S2e, f), consisting of pseudocapacitive anode and high-rate battery-type cathode. By using faradaic reaction on both cathode and anode, the designed PBH is able to deliver much enhanced energy density than those of EDLC-based hybrid supercapacitors. Besides, the assembly of PBH sodium-ion capacitor (PBH-SIC), using high-performance pseudocapacitive anode without presodiation treatments, is able to achieve an energy density over 100 Wh kg^{-1}.

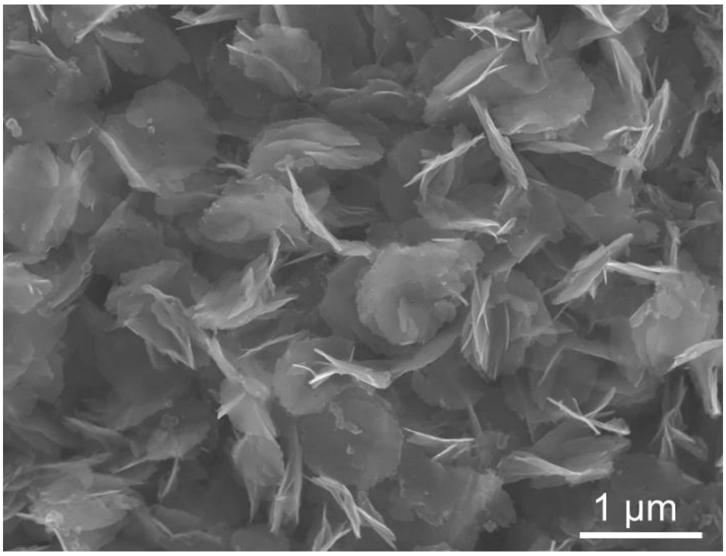

Fig. S3 SEM image of as-synthesized FeVO flowers. The BET specific surface area of FeVO flowers is 34.3 m^2 g^{-1}.

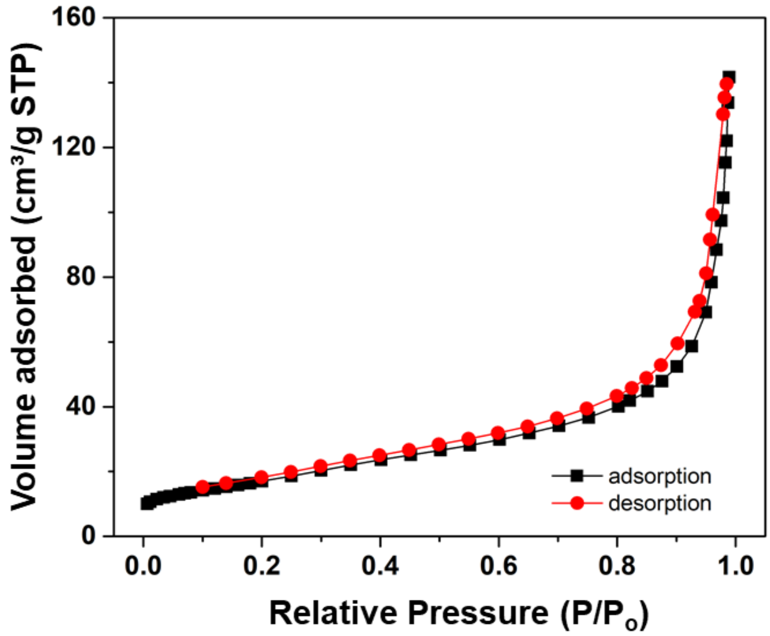

Fig. S4 Nitrogen adsorption/desorption isotherm of FeVO UNSs. The FeVO UNSs have a specific Brunauer–Emmett–Teller (BET) surface area of 62.6 m^2 g^{-1}.

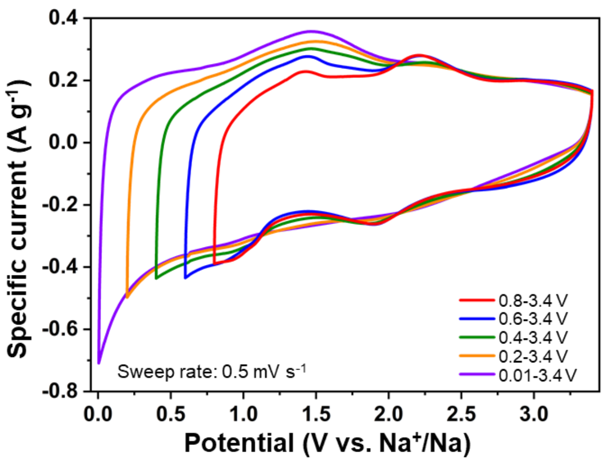

Fig. S5 Tuning the cut-off potentials of the FeVO UNSs anode. CV curves of FeVO UNSs anode in different potential ranges. The sweep rate is 0.5 mV s^{-1}.

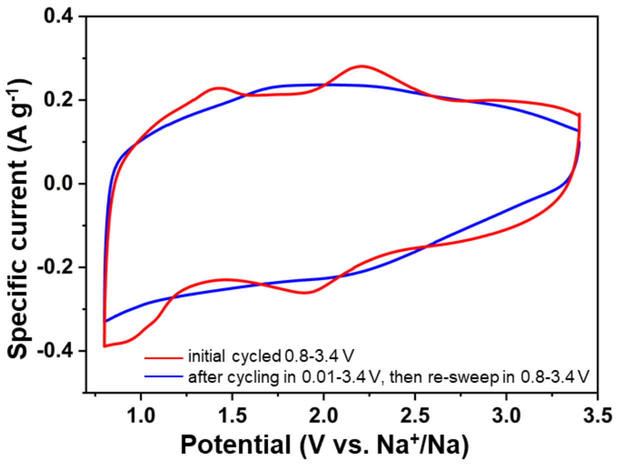

Fig. S6 Comparison of the CV curves of FeVO UNSs anode initial cycled in 0.8-3.4 V and re-sweep after cycled in 0.01-3.4 V. The sweep rate is 0.5 mV s^{-1}. After cycled in 0.01-3.4 V, we re-cycled the coin cell in the range of 0.8-3.4 V again. But the CV curve only shows a smooth rectangular shape without any redox peaks, which is owing to an irreversible amorphous transformation of conversion reaction.

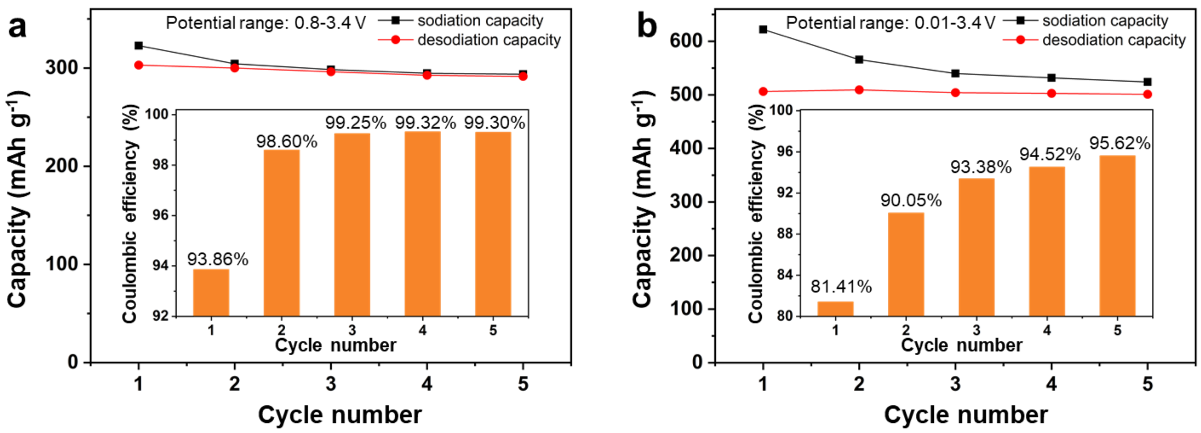

Fig. S7 (**a**) Capacity of of FeVO UNSs in the initial 5 cycles, cycled at 0.1 A g^{-1} in 0.8-3.4 V, the inset is the related coulombic efficiency. (**b**) Capacity of of FeVO UNSs in the initial 5 cycles, cycled at 0.1 A g^{-1} in 0.0.1-3.4 V, the inset is the related coulombic efficiency.

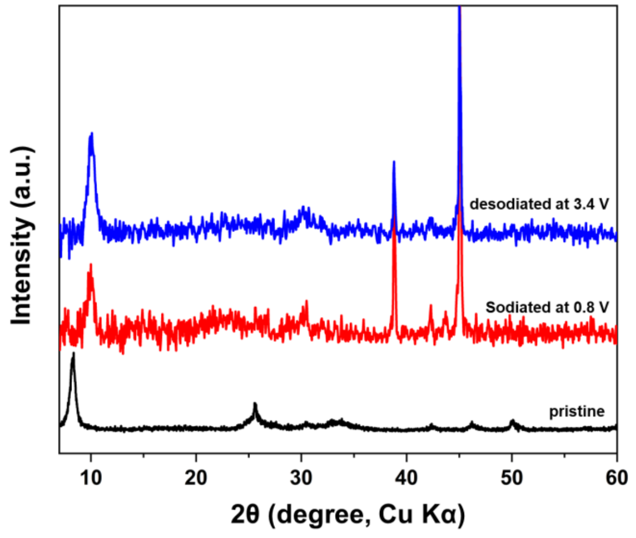

Fig. S8 Ex-situ XRD patterns of FeVO UNSs after sodiated at 0.8 V and desodiated at 3.4 V

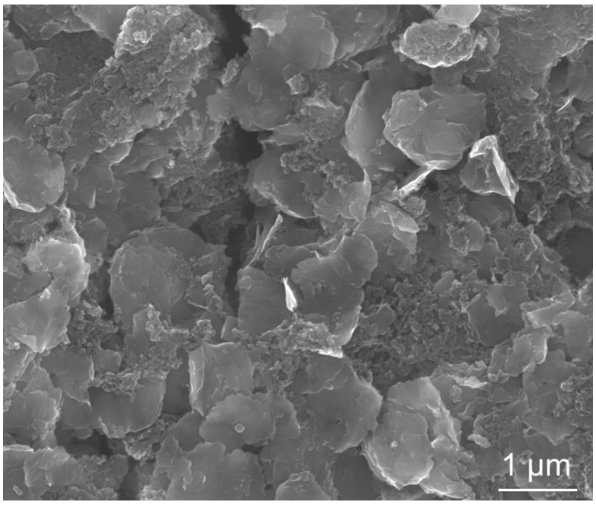

Fig. S9 SEM image of the pristine FeVO UNSs anode (before cycling test)

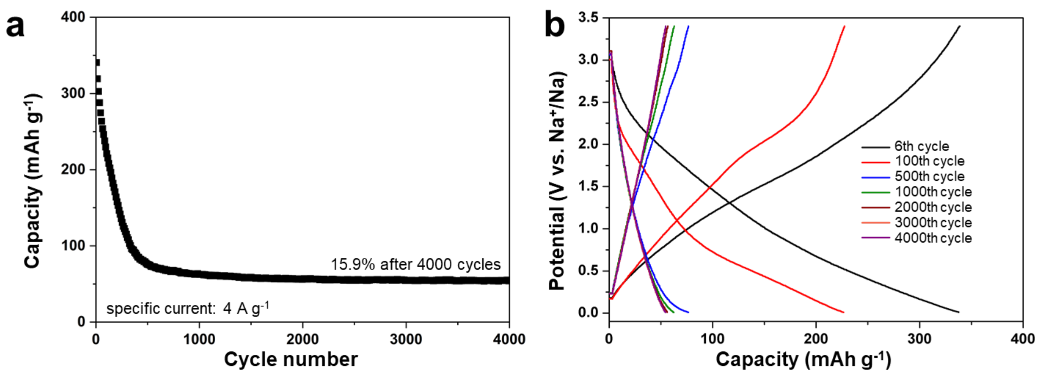

Fig. S10 Cycling performance (**a**) and related charge-discharge profiles (**b**) of FeVO UNSs anode tested at 4 A g^{-1} in 0.01-3.4 V, respectively. The FeVO anode shows a sodiation capacity of 340 mAh g^{-1}, and it quickly decays to 76 mAh g^{-1} after 500 cycles and 54 mAh g^{-1} after 4,000 cycles, respectively. The related charge-discharge curves show rapid loss of redox reaction.

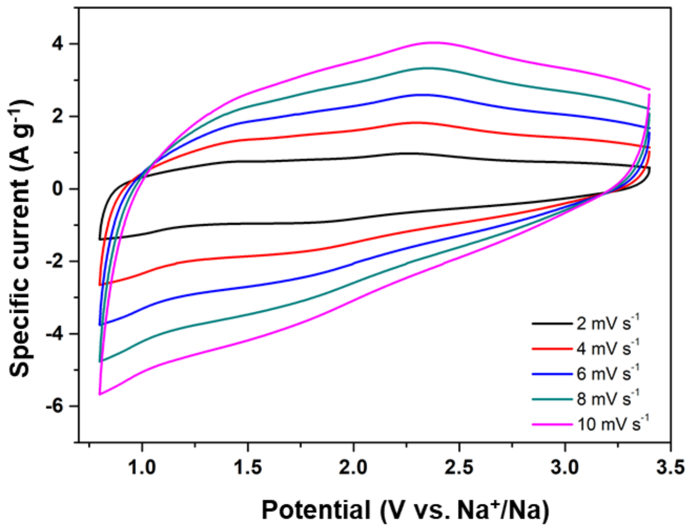

Fig. S11 CV curves of FeVO UNSs anode in 0.8-3.4 V at different sweep rates from 2 to 10 mV s^{-1}

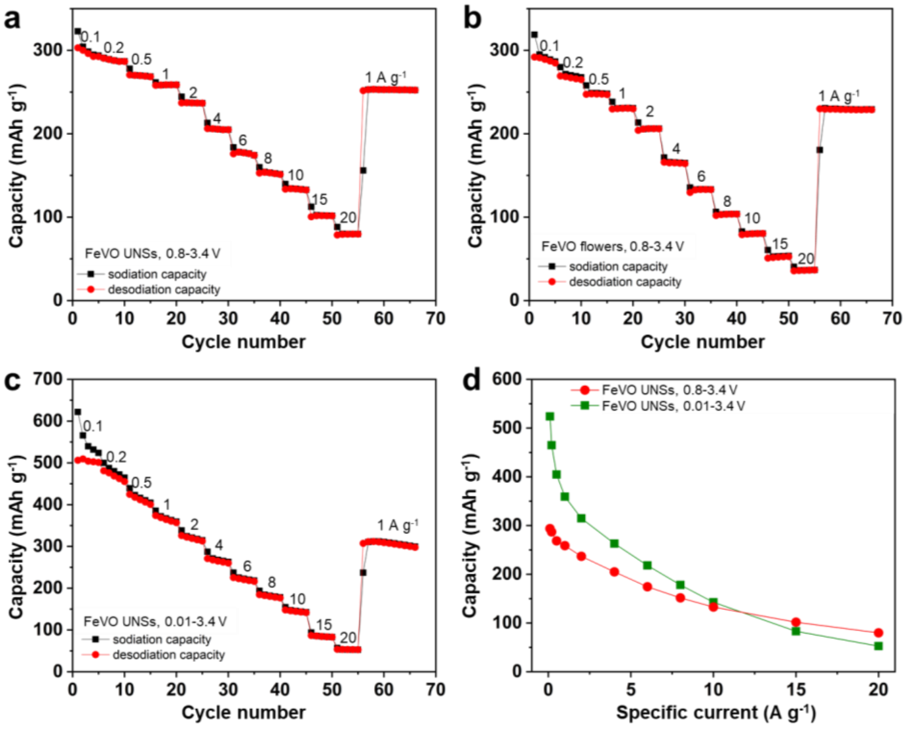

Fig. S12 (**a**) Rate performance of the FeVO UNSs cycled in 0.8-3.4 V. (**b**) Rate performance of the FeVO flowers cycled in 0.8-3.4 V. (**c**) Rate performance of the FeVO UNSs cycled in 0.01-3.4 V. (**d**) Rate capability of FeVO UNSs cycled in 0.8-3.4 V and 0.01-3.4 V

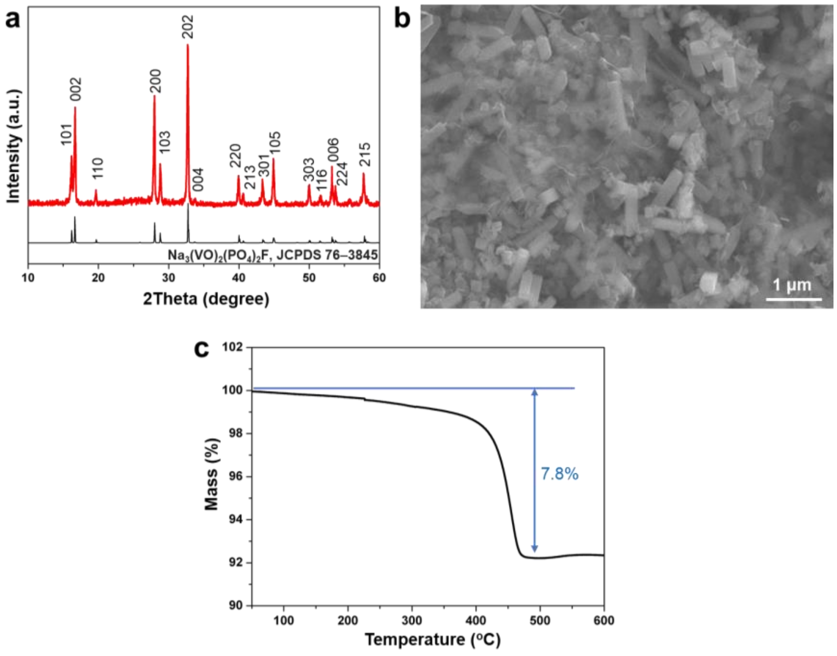

Fig. S13 XRD pattern (**a**), SEM image (**b**) and TG curves (**c**) of NVOPF/rGO composites. The XRD result confirms the sample is tetragonal P42/mnm phase of $Na_3(VO)_2(PO_4)_2F$ (JCPDS 76–3845). SEM image shows NVOPF nanorods are wrapped by rGO sheets. The graphene content of NVOPF/rGO composites is ~7.8 wt%.

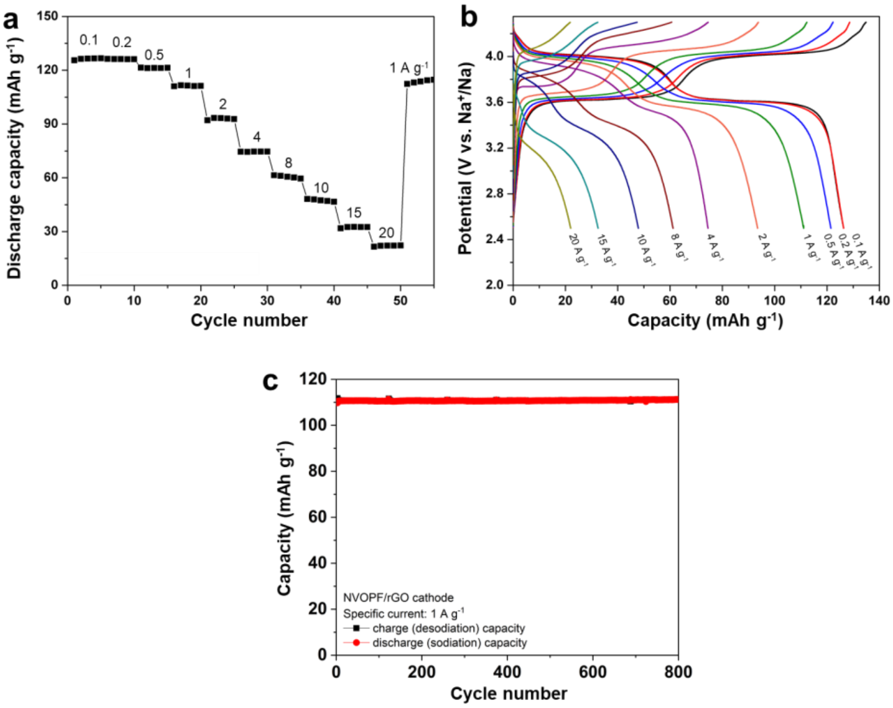

Fig. S14 Rate capability (**a**), related charge-discharge curves at different specific currents (**b**) and cycling performance at the specific current of 1 A g^{-1} (**c**) of the NVOPF/rGO cathode, respectively. The initial desoditaion and sodiation capacity of the NVOPF/rGO is 135 and 126 mAh g^{-1}, respectively. The NVOPF/rGO cathode delivers a high capacity of 92 mAh g^{-1} at 2 A g^{-1}, 48 mAh g^{-1} at 10 A g^{-1} and 22 mAh g^{-1} at 20 A g^{-1}, indicating the excellent high-rate capability. The NVOPF/rGO cathode display a capacity retention of 99.4% after 800 cycles, at a specific current of 1 A g^{-1}.

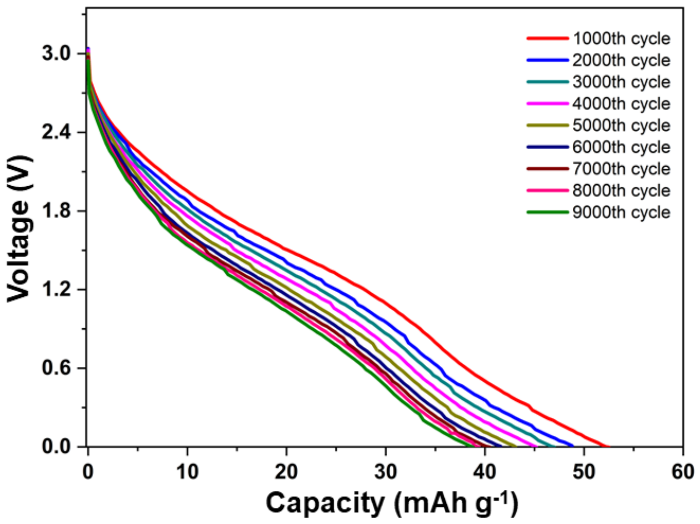

Fig. S15 Charge-discharge profiles of FeVO//NVOPF PBH-SIC at different cycles, while the specific current is 1.2 A g^{-1} (20 C)

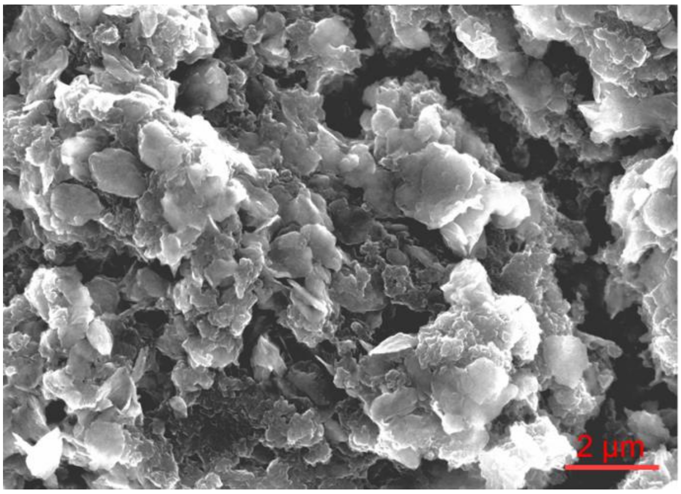

Fig. S16 SEM image of the FeVO UNSs anode from PBH-SIC after rate performance testing

Table S1 A survey of electrochemical properties of sodium storage anode materials

Anode materials	Reversible capacity (mAh g^{-1})	ICE (%)	Potential range (V *vs.* Na^+/Na)	Rate capability (mAh g^{-1})	Cycling stability (%)	Mass loading (mg cm^{-2})	Electrode composite	Electrolyte	Refs.
**FeVO UNSs**	**292 at 0.1 A g^{-1}**	**93.86**	**0.8-3.4 V**	**132 at 10 A g^{-1} 80 at 20 A g^{-1}**	**74.6%, 4000 cycles at 4 A g^{-1}**	**1.5-2.0**	**8:1:1**	**1 M $NaPF_6$ in DGDE**	**This work**
Mesoporous TiO_2/rGO	268 at 0.067 A g^{-1}	~30	0.01-3 V	104 at 6.7 A g^{-1}	18,000 cycles at 3.35 A g^{-1}	1.5-2	7:2:1	1 M $NaClO_4$ in $EC_{50}PC_{50}FEC_5$	[S1]
Nb_2O_5@C/rGO	285 at 0.025 A g^{-1}	31	0.01-3 V	109 at 3 A g^{-1}	500 cycles at 1.25 A g^{-1}	0.7	8:1:1	1 M $NaPF_6$ in $EC_{50}DMC_{50}FEC_5$	[S2]
Ti_2CT_x-MXene	200 at 0.02 A g^{-1}	65	0.1-3 V	63 at 5 A g^{-1}	100 cycles at 0.02 A g^{-1}	1	8:1:1	1 M $NaPF_6$ in $EC_{50}DEC_{50}$	[S3]
3D V_2CT_x-MXene frameworks	340 at 0.05 A g^{-1}	55.3	0.01-3 V	170 at 5 A g^{-1}	Capacity increased, 1000 cycles at 0.5 A g^{-1}	0.5-1	--	1 M $NaClO_4$ in $EC_{50}PC_{50}FEC_5$	[S8]
MoS_2 NS@NG	~275 at 0.1 A g^{-1}	~72	0.4-3 V	141 at 1.28 A g^{-1}	86%, 1000 cycles at 1 A g^{-1}	0.8-1	7:2:1	1 M $NaClO_4$ in $EC_{50}DEC_{50}FEC_{10}$	[S9]
Expandable Soft Carbon	188 at 0.02 A g^{-1}	67.6	0.01-2 V	114 at 1 A g^{-1}	300 cycles at 1 A g^{-1}	--	8:1:1	1 M $NaPF_6$ in $EC_{50}DEC_{50}$	[S10]
$NaTi_2(PO_4)_3$	101 at 0.133	79	1.5-3 V	67 at 6.65 A g^{-1}	80%, 1000	1.5	8:1:1	1 M $NaClO_4$ in	[S11]

in graphene networks	A g^{-1}				cycles at 1.33 A g^{-1}			$EC_{50}DEC_{50}$	
Hard carbon	361 at 0.02 A g^{-1}	86.1	0-2 V	--	93.4%, 100 cycles at 0.02 A g^{-1}	--	8:1:1	1 M $NaClO_4$ in $EC_{50}DEC_{50}$	[S12]
γ-Fe_2O_3@C	993 at 0.2 A g^{-1}	51.5	0.04-3 V	317 at 8 A g^{-1}	1400 cycles at 2 A g^{-1}	1	8:1:1	1 M $NaClO_4$ in $EC_{50}DEC_{50}$	[S13]
Anatase TiO_2 nanoparticles	217 at 0.05 A g^{-1}	56	0.01-3 V	129 at 4 A g^{-1}	89%, 600 cycles at 2 A g^{-1}	1.0	7:2:1	1 M $NaPF_6$ in DGDE	[S14]
Anatase TiO_2 nanoparticles	181 at 0.05 A g^{-1}	40	0.01-3 V	32 at 4 A g^{-1}	23.8%, 500 cycles at 0.1 A g^{-1}	1.0	7:2:1	1 M $NaPF_6$ in $EC_{50}DEC_{50}$	[S14]
Sb−C framework film	451 at 0.117 A g^{-1}	65	0-2.5 V	198 at 7.02 A g^{-1}	~100%, 5000 cycles at 3.51 A g^{-1}	1.3-1.65	--	1 M $NaClO_4$ in $PC_{100}FEC_5$	[S15]
P2-$Na_{0.66}[Li_{0.22}Ti_{0.78}]O_2$	116 at 0.016 A g^{-1}	75	0.4-2.5 V	62 at 0.212 A g^{-1}	75%, 2000 cycles at 0.212 A g^{-1}	--	6:3:1	1 M $NaClO_4$ in EC_{40}:DEC_{60}	[S16]
$NiCo_2O_4$ mesoporous NSs	670 at 0.1 A g^{-1}	68.4	0-3 V	170 at 3.2 A g^{-1}	72%, 100 cycles at 1.6 A g^{-1}	1.0	8:1:1	1 M $NaClO_4$ in $PC_{100}FEC_2$	[S17]

The abbreviation of DGDE, EC, PC, DMC, DEC, FEC represent diethylene glycol dimethyl ether ethylene carbonate, propylene carbonate, dimethyl carbonate, diethyl carbonate, fluoroethylene carbonate, respectively. ICE, initial coulombic efficiency. NG, nitrogen-doped graphene.

Table S2 Comparison between sodium storage in different pseudocapacitive anode materials and hybrid SICs.

Anode materials	Mesoporous TiO_2/rGO (Ref. [S1])	Nb_2O_5@C/rGO (Ref. [S2])	Ti_2CT_x-MXene (Ref. [S3])	FeVO UNSs (this work)
Reversible Capacity (mAh g^{-1})	189	285	190	292
ICE[a)]	~32%	~31%	~65%	93.86%
Potential range (V *vs.* Na^+/Na)	0.01-3 V	0.01-3 V	0.1-3 V	0.8-3.4 V
Cathode materials	activated carbon	activated carbon	$Na_2Fe_2(SO_4)_3$	$Na_3(VO)_2(PO_4)_2F$
Additional treatment	electrochemical pre-sodiation	electrochemical pre-sodiation	overcapacity cathode	No need
C/A ratio[b)]	5:1	4:1	4:1	2.5:1
Energy capability[c)]	64 Wh kg^{-1} at 56 W kg^{-1}	76 Wh kg^{-1} at 80 W kg^{-1}	65 Wh kg^{-1} at 72 W kg^{-1}	126 Wh kg^{-1} at 91 W kg^{-1}
Power capability[c)]	26 Wh kg^{-1} at 1.3 kW kg^{-1}	38 Wh kg^{-1} at 1.3 kW kg^{-1}	52 Wh kg^{-1} at 0.288 kW kg^{-1}	87 Wh kg^{-1} at 1.3 kW kg^{-1}
Longevity of SIC	90% after 10000 cycles	~65% after 3000 cycles	~100% after 100 cycles	67% after 10000 cycles
Operation voltage range of SIC	1-3.8 V	1-4.3 V	0.1-3.8 V	0-3.3 V

Supplementary References

[S1] Z. Le, F. Liu, P. Nie, X. Li, X. Liu et al., Pseudocapacitive sodium storage in mesoporous single-crystal-like TiO_2–graphene nanocomposite enables high-performance sodium-ion capacitors. ACS Nano **11**(3), 2952-2960 (2017).

https://doi.org/10.1021/acsnano.6b08332

[S2] E. Lim, C. Jo, M.S. Kim, M.-H. Kim, J. Chun et al., High-performance sodium-ion hybrid supercapacitor based on Nb_2O_5@carbon core–shell nanoparticles and reduced graphene oxide nanocomposites. Adv. Funct. Mater. **26**(21), 3711-3719 (2016). https://doi.org/10.1002/adfm.201505548

[S3] X. Wang, S. Kajiyama, H. Iinuma, E. Hosono, S. Oro et al., Pseudocapacitance of mxene nanosheets for high-power sodium-ion hybrid capacitors. Nat. Commun. **6**(1), 6544 (2015). https://doi.org/10.1038/ncomms7544

[S4] B. Li, J. Zheng, H. Zhang, L. Jin, D. Yang et al., Electrode materials, electrolytes, and challenges in nonaqueous lithium-ion capacitors. Adv. Mater. **30**(17), 1705670 (2018). https://doi.org/10.1002/adma.201705670

[S5] K. Naoi, S. Ishimoto, Y. Isobe, S. Aoyagi. High-rate nano-crystalline $Li_4Ti_5O_{12}$ attached on carbon nano-fibers for hybrid supercapacitors. J. Power Sources **195**(18), 6250-6254 (2010). https://doi.org/10.1016/j.jpowsour.2009.12.104

[S6] P. F. R. Ortega, G. A. dos Santos Junior, L. A. Montoro, G. G. Silva, C. Blanco et al., $LiFePO_4$/mesoporous carbon hybrid supercapacitor based on litfsi/imidazolium ionic liquid electrolyte. J. Phys. Chem. C **122**(3), 1456-1465 (2018). https://doi.org/10.1021/acs.jpcc.7b09869

[S7] J. Ding, W. Hu, E. Paek, D. Mitlin. Review of hybrid ion capacitors: From aqueous to lithium to sodium. Chem. Rev. **118**(14), 6457-6498 (2018). https://doi.org/10.1021/acs.chemrev. 8b00116

[S8] M.-Q. Zhao, X. Xie, C.E. Ren, T. Makaryan, B. Anasori et al., Hollow MXene spheres and 3D macroporous mxene frameworks for Na-ion storage. Adv. Mater. **29**(37), 1702410 (2017). https://doi.org/10.1002/adma.201702410

[S9] X. Xu, R. Zhao, W. Ai, B. Chen, H. Du et al., Controllable design of MoS_2 nanosheets anchored on nitrogen-doped graphene: toward fast sodium storage by tunable pseudocapacitance. Adv. Mater. **30**(27), 1800658 (2018). https://doi.org/10.1002/adma.201800658

[S10] W. Luo, Z. Jian, Z. Xing, W. Wang, C. Bommier, et al., Electrochemically expandable soft carbon as anodes for Na-ion batteries. ACS Cent. Sci. **1**(9), 516-522 (2015). https://doi.org/10.1021/acscentsci.5b00329

[S11] C. Wu, P. Kopold, Y.-L. Ding, P.A. van Aken, J. Maier et al., Synthesizing porous $NaTi_2(PO_4)_3$ nanoparticles embedded in 3d graphene networks for high-rate and long cycle-life sodium electrodes. ACS Nano **9**(6), 6610-6618 (2015). https://doi.org/10.1021/acsnano. 5b02787

[S12] L. Xiao, H. Lu, Y. Fang, M.L. Sushko, Y. Cao et al., Low-defect and low-porosity hard carbon with high coulombic efficiency and high capacity for practical sodium ion battery anode. Adv. Energy Mater. **8**(20), 1703238 (2018). https://doi.org/10.1002/aenm.201703238

[S13] N. Zhang, X. Han, Y. Liu, X. Hu, Q. Zhao et al., 3D porous γ-Fe_2O_3@c nanocomposite as high-performance anode material of Na-ion batteries. Adv. Energy Mater. **5**(5), 1401123 (2015). https://doi.org/10.1002/aenm.201401123

[S14] Z.-L. Xu, K. Lim, K.-Y. Park, G. Yoon, W.M. Seong et al., Engineering solid electrolyte interphase for pseudocapacitive anatase TiO_2 anodes in sodium-ion batteries. Adv. Funct. Mater. **28**(29), 1802099 (2018). https://doi.org/10.1002/adfm.201802099

[S15] B. Kong, L. Zu, C. Peng, Y. Zhang, W. Zhang et al., Direct superassemblies of freestanding metal–carbon frameworks featuring reversible crystalline-phase transformation for electrochemical sodium storage. J. Am. Chem. Soc. **138**(50), 16533-16541 (2016). https://doi.org/10.1021/jacs.6b10782

[S16] Y. Wang, X. Yu, S. Xu, J. Bai, R. Xiao et al., A zero-strain layered metal oxide as the negative electrode for long-life sodium-ion batteries. Nat. Commun. **4**(1), 2365 (2013). https://doi.org/10.1038/ncomms3365

[S17] L. Peng, P. Xiong, L. Ma, Y. Yuan, Y. Zhu et al., Holey two-dimensional transition metal oxide nanosheets for efficient energy storage. Nat. Commun. **8**(1), 15139 (2017). https://doi.org/10.1038/ncomms15139
